# Development and Validation of a Versatile UPLC-PDA Method for Simultaneous Determination of Paracetamol, Tizanidine, Aceclofenac, and Nimesulide in Their New Combinations

**DOI:** 10.1155/2018/7463914

**Published:** 2018-05-14

**Authors:** Sami Bawazeer, Khalid M. Badr El-Din, Ahmed M. Abdel-Megied

**Affiliations:** ^1^Pharmaceutical Analysis Unit, Umm Al-Qura University, Mecca, Saudi Arabia; ^2^Analytical Chemistry Department, Faculty of Pharmacy, Minya University, El-Minya, Egypt; ^3^Pharmaceutical Chemistry Department, Faculty of Pharmacy, Deraya University, Minya, Egypt; ^4^Pharmaceutical Analytical Chemistry Department, Faculty of Pharmacy, Kafrelsheikh University, Kafr El Sheikh, Egypt

## Abstract

A simple, rapid, and validated UPLC method was developed for the simultaneous quantitation of paracetamol (PAR), tizanidine (TIZ), aceclofenac (ACF), and nimesulide (NIM) either in pure forms or in their different tablet dosage forms. Chromatographic separation was attained on an ACQUITY UPLC™ BEH C18 column (100 mm × 2.1 mm, 1.7 *μ*m) with a mobile phase consisting of 20 mM phosphate buffer (pH 7.0) : acetonitrile in the proportion (60 : 40 *v*/*v*) isocratically pumped at a flow rate of 1.25 mL·min^−1^, and detection was monitored at 305 nm. All analytes were separated simultaneously at a retention time (*t*_r_) of 1.42, 2.31, 3.63, and 5.62 min for PAR, TIZ, ACF, and NIM, respectively, with a total run time less than 6.0 min. The proposed method was validated according to ICH guidelines with respect to accuracy, precision, linearity, limit of detection, limit of quantitation, and robustness. Linearity was obtained over a concentration range of 81.25–487.5, 0.5–3.5, 25–150, and 25–150 *µ*g·mL^−1^ for PAR, TIZ, ACF, and NIM, respectively. The development method can be successfully employed in QC laboratories for the routine analysis of the investigated drugs in their new combination.

## 1. Introduction

**Figure 1:**
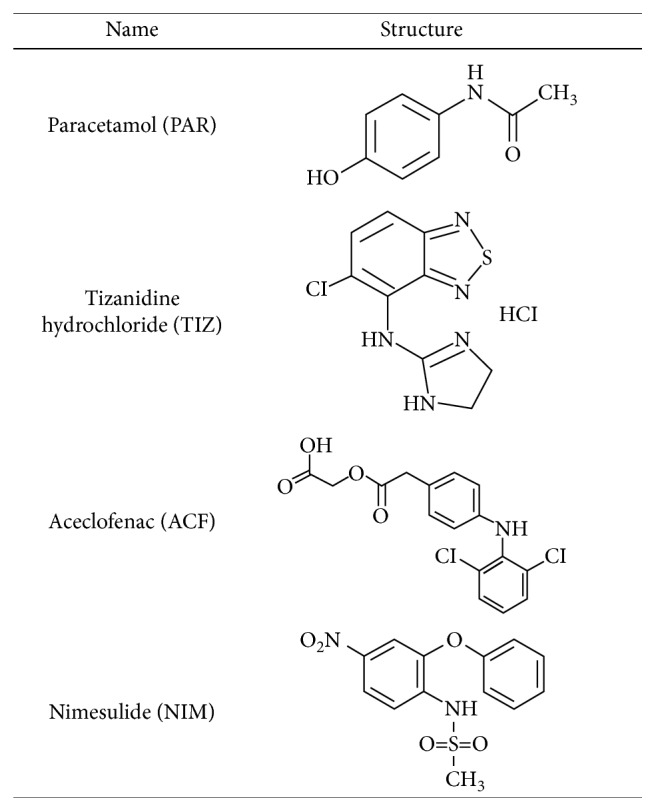
The chemical structures of paracetamol (PAR), tizanidine (TIZ), aceclofenac (ACF), and nimesulide (NIM).

Anybody during his lifetime would probably suffer from acute pain. Pain is considered a warning for a certain danger and in the same time as a reminder to protect injured limbs and tissues during the healing process [1]. Nowadays, combination therapy using nonsteroidal anti-inflammatory drugs (NSAIDs) is the favorable mainstay of pain relief due to its synergistic effects, multiple actions, quick relief, and patient acceptance [2]. Paracetamol (PAR) is a potent analgesic and antipyretic; chemically, it is *N*-(4-hydroxyphenyl)acetamide [3]. There has been a trend over recent years for combining NSAIDs with paracetamol (PAR) for the management of acute postoperative pain, but the therapeutic superiority of the combination over either drug alone remains controversial [4, 5]. It was noted that PAR/NSAID combinations showed superior pain relief over PAR alone in 5 of 7 studies but over an NSAID alone in only 2 of 4 studies [6]. Nimesulide (NIM) is a derivative of *p*-nitrophenyl methanesulfonamide which belongs to selective COX-2 inhibitors, and it has a potent analgesic and anti-inflammatory activity that could be used for the treatment of various inflammatory processes; chemically, it is *N*-(4-nitro-2-phenoxyphenyl)methanesulfonamide [7, 8]. Aceclofenac (ACF) is a phenylacetic acid derivative with anti-inflammatory and potent analgesic properties in the treatment of rheumatoid arthritis and osteoarthritis with an improved gastrointestinal tolerance; chemically, it is 2-[2-[2-(2,6-dichloroanilino)phenyl]acetyl]oxyacetic acid [9]. Tizanidine hydrochloride (TIZ) is a skeletal muscle relaxant that acts centrally in the treatment of spasticity due to multiple sclerosis and spinal cord injury; chemically, it is 5-chloro-*N*-(4,5-dihydro-1H-imidazole-2yl)-2,1,3-benzo-thiadiazol-4-amine hydrochloride [7]. The structure formulae of the investigated drugs are shown in Figure 1. Various fixed-dose combinations of NSAIDs are available in the global market, and they vary in the amount of ingredients such as PAR, NIM (Nicip plus®) and/or ACF, TIZ (Zerodol MR®) combinations with faster onset and longer duration of analgesic and antipyretic effects than either drug alone [10, 11].

The literature review reveals a number of reported methods for the determination of cited drugs either separately or in the presence of others such as UV-Vis spectrophotometry [12–14], HPLC [12, 15–18], HPTLC [19], CE [20], LC-MS/MS [21], and electrochemical method [22]. To the best of the author's knowledge, there is no single chromatographic method reported to cover the analysis of all mentioned drugs simultaneously in their tablets.

The present work is aimed at developing a fully validated highly sensitive methodology according to ICH guidelines for the simultaneous determination of all the studied drugs in their pharmaceutical dosage forms using an isocratic chromatographic mode with an analysis time less than six minutes. The proposed method was successfully employed for routine quality control in the new marketed tablets.

## 2. Experimental

### 2.1. Chemicals and Reagents

PAR, TIZ, ACF, and NIM were purchased from Qualigens Fine Chemicals Ltd. (Mumbai, India). Acetonitrile (HPLC grade) was supplied by Labscan Ltd. (Dublin, Ireland); orthophosphoric acid (85%) was supplied by Adwic-El Nasr Pharmaceutical Chemicals Co. (Cairo, Egypt). Dipotassium hydrogen phosphate of AR grade was purchased from S D Fine Chemicals (Mumbai, India). Ultrapure water with resistivity >18 MΩ·cm^−1^ at 25°C and TOC < 5 ppb was obtained from the Milli-Q UF-Plus system (Millipore, USA).

### 2.2. Pharmaceutical Formulations

The following dosage forms were analyzed: Nicip plus tablets from Cipla Pharmaceuticals (Maharashtra, India) claimed to contain 100 mg of nimesulide and 325 mg of paracetamol per tablet. Zerodol MR tablets from IPCA Laboratories Ltd. (Maharashtra, India) claimed to contain 100 mg of aceclofenac and 2 mg of tizanidine per tablet. Zerodol P® tablets from IPCA Laboratories Ltd. (Maharashtra, India) were labeled to contain 100 mg of aceclofenac and 500 mg of paracetamol per tablet. Nobel MR® tablets from Pharma Force Lab (Gondpur, India) were labeled to contain 100 mg of nimesulide, 325 mg of paracetamol, and 2 mg of tizanidine. These tablets are commercially available in the Indian market and were purchased from public pharmacies in India with help of our colleagues.

### 2.3. Instrumentation

- All chromatographic analyses were performed using a Waters ACQUITY® UPLC-PDA H-class system (Milford, MA, USA), which included an autosampler, an ultrahigh-performance quaternary pump, a column heater, and a tunable ultraviolet (TUV) detector. Data acquisitions were carried out using Empower™ 2.0 software. An ultrasonicator Model L-7612 (Merck, USA) was used.
- A Jenway digital pH meter (Staffordshire, UK) was used.

### 2.4. Chromatographic Conditions

Chromatographic separations were achieved on an ACQUITY UPLC BEH C18 column (100 mm × 2.1 mm, 1.7 *μ*m) with a mobile phase consisting of phosphate buffer (20 mM, pH 7.0) : acetonitrile in the proportion (60 : 40 *v*/*v*) isocratically pumped at a flow rate of 1.25 mL·min^−1^, and detection was monitored at 305 nm at room temperature. Filtration of the mobile phase was performed using a 0.45 *μ*m Millipore membrane filter (Billerica, MA). The injection volumes were 2 *μ*L. The peak areas were integrated automatically using Empower 2.0 software.

### 2.5. Standard Solutions

Primary stock solutions of 1.0 mg·mL^−1^ PAR, TIZ, ACF, and NIM were prepared using the optimized mobile phase separately with the aid of ultrasonic bath. Freshly prepared working solutions were performed by dilution of the primary stock solutions with the same solvent to obtain a concentration of 4875, 35, 1500, and 1750 *μ*g·mL^−1^ for PAR, TIZ, ACF, and NIM, respectively. In 10 mL volumetric flasks, the prepared working standard solutions of PAR, NIM, ACF, and TIZ were diluted in an appropriate volume with the mobile phase. All solutions were kept in the refrigerator (2–8°C) for one week without alteration.

### 2.6. Procedures

#### 2.6.1. Construction of Calibration Graphs

Aliquot volumes of each working standard solution were accurately measured and transferred into a series of 10 mL volumetric flasks so that the final concentration was in the range of 81.25–487.5 *µ*g·mL^−1^ for PAR, 0.5–3.5 *µ*g·mL^−1^ for TIZ, 25–150 *µ*g·mL^−1^ for ACF, and 25–150 *µ*g·mL^−1^ for NIM. 2 *μ*L was injected in triplicate and eluted with the mobile phase under the optimum chromatographic conditions. The calibration graphs were constructed by plotting the peak area versus the corresponding concentration, and the regression equation was computed.

#### 2.6.2. Assay of PAR, TIZ, ACF, and NIM in Laboratory-Prepared Mixtures

Synthetic mixtures were prepared through mixing different known amounts of working standard solutions with those of the other components in different ratios including those of commercial tablets to verify the precision of the proposed method for the analysis of such mixtures.

#### 2.6.3. Assay of PAR, TIZ, ACF, and NIM in Their Tablets

Ten tablets of each dosage form were weighed carefully and crushed completely to a fine powder. An accurately weighed amount equivalent to 325, 2, 100, and 500 mg of PAR, TIZ, ACF, and NIM, respectively, was transferred to a 100 mL volumetric flask and made up to 80.0 mL with methanol. All solutions were sonicated in water bath for 15 min, vortex mixed for 5 min, diluted to the mark with methanol, and finally filtered through a Millipore filter (0.45 *µ*m pore size), and aliquots of all solutions were analyzed as appropriate (as discussed in Section 2.6.1). The nominal contents of the tablets were calculated using the previously plotted calibration graph or the corresponding regression equation.

#### 2.6.4. Validation

System suitability tests were performed by injecting different concentrations of the working standard solutions for all drugs under investigation, and the separation factor was monitored throughout the validation process. Precision and intermediate precision (for three consecutive days) were checked using six replicate injections of all drugs, and peak areas were measured for which the relative standard deviation was computed. Limit of detection and limit of quantitation were determined at a signal-to-noise ratio for which LOD = 3.3 *σ*/*S* and LOQ = 10 *σ*/*S*, where *σ* is the standard deviation of the intercept and *S* is the slope derived from the calibration curve [23]. Linearity of the detector response was applied by preparing six calibration sample solutions starting with LOQ concentration. Each set of solutions was prepared in triplicates and analyzed for three successive days, and the % RSD and Y-intercept of the calibration curves were computed. The samples were stored in a tightly closed volumetric flask at 4°C temperature and analyzed after 48 hours.

#### 2.6.5. Procedures for the Standard Addition Method (Checking Accuracy and Specificity)

Fixed portions of working dosage forms of solutions were quantitatively transferred to a 10.0 mL volumetric flask, and serial portions of working standard solutions of cited drugs were added to each flask. The solutions were mixed well and then completed with the used solvent to the volume, and chromatographic procedures were followed as mentioned in Section 2.6.1.

## 3. Results and Discussion

### 3.1. Method Development and Optimization of LC Conditions

**Figure 2:**
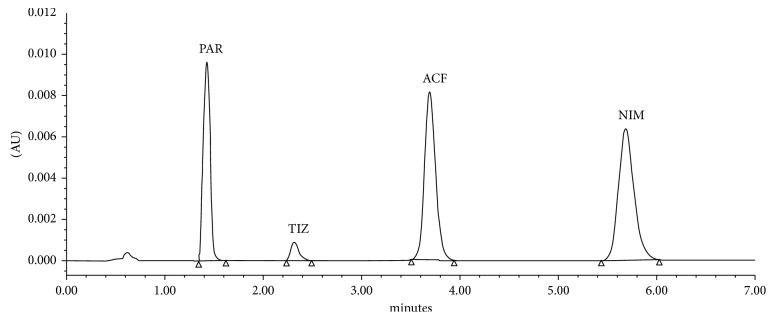
Typical UPLC chromatogram for simultaneous separation of PAR, TIZ, ACF, and NIM. Chromatographic conditions are as follows: ACQUITY UPLC BEH C18 column (100 mm × 2.1 mm, 1.7 *μ*m); mobile phase consisting of acetonitrile : 20 mM phosphate buffer adjusted to pH 7.0 using dipotassium hydrogen phosphate (40 : 60 *v*/*v*); flow rate 1.25 mL·min^−1^; injection volume 2.0 *μ*L; and PDA detection at 305 nm.

This is the first method that applies UPLC rather than HPLC for the determination of PAR, TIZ, ACF, and NIM simultaneously in one single run with many advantages as UPLC operates at much higher pressures, improved resolution, and fewer consumables. Different columns were tried including an ACQUITY UPLC BEH C18 column (100 mm × 2.1 mm, 1.7 *μ*m), an ACQUITY UPLC BEH C8 column (100 mm × 2.1 mm, 1.7 *μ*m), and a Zorbax SB-CN column (50 × 4.6 mm, 1.8 *μ*m). The C8 and CN columns showed a bad resolution for PAR, tailing between ACF and TIZ, and long retention time for NIM as well. After several trials, the C18 column was the most suitable one with high resolution and produced symmetrical peaks and reasonable time of analysis less than 6 min. Preliminary trials with mobile phase compositions in different ratios between the organic modifier and aqueous phase at pH 2–7 were performed in an isocratic elution mode. The best peak shape was obtained by the use of 20 mM phosphate buffer and adjusted to pH 7.0 with dipotassium hydrogen phosphate and acetonitrile, in the proportion (60 : 40 *v*/*v*). Acetonitrile was selected as an organic constituent of the mobile phase to reduce the retention time, and the buffer was preferred to reduce the peak asymmetry and to achieve a good peak shape. Adjusting the flow rate at 1.25 mL·min^−1^ was crucial for the proposed method to enhance the resolution between the four peaks. Applying the flow rate more than that would increase the back pressure of the UPLC system more than 400 psi is not favorable. The optimum wavelength for detection was 305 nm at which much better detection response for all analytes was achieved. Under the described conditions, the analytes' peaks were well defined, resolved, and free from tailing at 1.42, 2.31, 3.63, and 5.62 for PAR, TIZ, ACF, and NIM, respectively, as shown in Figure 2. To determine the linearity of HPLC detection response, calibration standard solutions of all drugs were prepared as described in the text. A linear correlation was obtained between the peak area versus the concentration of each drug. The characteristic parameters for regression equations of the proposed UPLC method are given in Table 1.

### 3.2. System Suitability

The U.S. Pharmacopeia (USP) [24] states that system suitability tests are an integral part of liquid chromatographic methods. They are used to verify that the resolution and reproducibility of the chromatographic system are adequate for the analysis to be done. Parameters including resolution (*R*), peak symmetry, capacity factor (*K*′), and height equivalent theoretical plates (*N*) were calculated as shown in Table 2.

### 3.3. Method Validation

The proposed UPLC method was validated according to ICH guidelines [25] with respect to parameters such as linearity, LOD, LOQ, precision, accuracy, specificity, and robustness.

#### 3.3.1. Linearity, LOD, and LOQ

Under the above-described experimental conditions, the linearity of the proposed method was investigated by plotting the peak areas of PAR, TIZ, ACF, and NIM versus the concentration of standard drugs. Linear regression equations are summarized as

(1) $$\begin{array}{c} \begin{array}{l} y=84.922x+958.16\;\mathrm{for}\;\mathrm{PAR},\\ y=1500.4x+89.248\;\mathrm{for}\;\mathrm{TIZ},\\ y=366.72x+563.8\;\mathrm{for}\;\mathrm{ACF,}\\ y=412.42x-1423.8\;\mathrm{for}\;\mathrm{NIM}. \end{array} \end{array}$$

LOD and LOQ were also estimated. All results are summarized in Table 1.

#### 3.3.2. Accuracy and Precision

The standard addition technique was performed to check the accuracy of the proposed method in which standard solutions of all cited drugs at different concentrations were added to the previously analyzed tablet samples. Furthermore, by applying the general analytical procedures, the total contents of all drugs were obtained. It was found that the obtained percentage recoveries were in the range of 97.6–102.33 as shown in Table 3, while precision was checked by analyzing standard solutions at three different concentration levels. Intraday precision was performed at three different times within the same day, while interday assays were applied at three successive days. The obtained results were expressed as percentage recovery, and relative standard deviations indicated the high accuracy and precision of the proposed UPLC method as shown in Table 4.

#### 3.3.3. Robustness

It was studied to measure the reliability of the developed UPLC method by deliberate variations in the optimized parameters such as the change in temperature and pH of the buffer solution as illustrated in Table 5.

### 3.4. Analysis of Laboratory-Prepared Mixtures

The results obtained by application of the proposed method for some suggested laboratory-prepared binary mixtures are shown in Table 6. As can be seen, the percentage recoveries in all cases were satisfactory, and the relative standard deviation value for both drugs did not exceed 2, indicating good accuracy and quality control applicability.

### 3.5. Specificity

It was studied by analyzing the synthetic mixtures for all studied drugs. The result showed good resolution and the absence of interference from other excipients as shown in Table 3.

## 4. Conclusion

A highly sensitive and an accurate UPLC method was developed for the simultaneous determination of PAR, TIZ, ACF, and NIM either in pure forms or in their different tablet dosage forms. With respect to analysis time, the proposed method has a distinct advantage for run time less than 6 min when compared with other previously reported methods. The proposed method, by virtue of its high sensitivity, could be performed in QC laboratories suitable for the routine analysis.

## Figures and Tables

**Table 1:** Analytical parameters for cited drugs by the proposed UPLC method.

Parameter	PAR	TIZ	ACF	NIM
Linear range (*µ*g·mL^−1^)	81.25–487.5	0.5–3.5	25–150	25–150
*a* (intercept)	958.16	89.25	563.80	−1423.8
*S*_*a*_ (standard deviation of the intercept)	146.30	17.36	323.92	244.29
*b* (slope)	84.92	1500.4	366.72	412.42
*S*_*b*_ (standard deviation of the slope)	0.47	7.98	3.41	2.362
*r* (correlation coefficient)	0.9999	0.9999	0.9997	0.9999
*r* ^2^ (determination coefficient)	0.9998	0.9998	0.9994	0.9998
SD of residuals (Sy.x)	174.21	22.17	338.72	323.91
LOD (limit of detection, *µ*g·mL^−1^)	5.68	0.03	2.92	1.96
LOQ (limit of quantitation, *µ*g·mL^−1^)	17.22	0.11	8.83	5.96

**Table 2:** Analytical parameters of system suitability tests for the proposed UPLC method.

Parameter	Reference value	PAR^∗^	TIZ^∗^	ACF^∗^	NIM^∗^
Flow rate (mL/min)	—	1.25			
Retention time (min)	—	1.42	2.32	3.63	5.63
Resolution (*R*)	*R* > 1.5	—	7.16	4.81	9.21
*K*′ (column capacity)	>2	5.48	9.55	15.51	24.59
Symmetry	—	1.24	1.40	1.18	1.18
Tailing factor (*T*)	≤2	1.22	1.38	1.16	1.21
*N* (column efficiency)	≥2000	2979.69	3036.93	5090.82	6074.17
HETP	=*L*/*N*	0.033	0.032	0.019	0.016

^∗^Mean of three determinations.

**Table 3:** Standard addition method for the calculation of percentage of studied drugs in their pharmaceutical dosage forms using the UPLC method.

Mixture number	Amount taken (*µ*g·mL^−1^)			Amount added (*µ*g·mL^−1^)			Amount found (*µ*g·mL^−1^)			% recovery^∗^		
*Nicip plus tablets*												
	NIM	PAR		NIM	PAR		NIM	PAR		NIM	PAR	
1	50.0	162.5		25.0	81.25		74.21	243.98		98.95	100.94	
2	50.0	162.5		50.0	162.5		98.01	323.01		98.01	99.39	
3	50.0	162.5		75.0	243.75		125.69	405.34		100.55	99.78	
*Zerodol MR tablets*												
	ACF	TIZ		ACF	TIZ		ACF	TIZ		ACF	TIZ	
1	50.0	1.0		25.0	1.0		76.19	1.97		101.59	98.50	
2	50.0	1.0		50.0	1.5		99.22	2.53		99.22	101.20	
3	50.0	1.0		75.0	2.0		124.14	2.96		99.31	98.67	
*Zerodol P tablets*												
	ACF	PAR		ACF	PAR		ACF	PAR		ACF	PAR	
1	50.0	162.5		25.0	81.25		76.45	244.10		101.93	100.14	
2	50.0	162.5		50.0	162.5		101.82	325.88		101.82	100.27	
3	50.0	162.5		75.0	243.75		124.44	404.39		99.55	99.54	
*Nobel MR tablets*												
	NIM	PAR	TIZ	NIM	PAR	TIZ	NIM	PAR	TIZ	NIM	PAR	TIZ
1	50.0	162.5	1.0	25.0	81.25	1.0	75.09	242.99	1.99	100.12	99.69	99.50
2	50.0	162.5	1.0	50.0	162.5	1.5	97.99	323.89	2.44	97.99	99.66	97.60
3	50.0	162.5	1.0	75.0	243.75	2.0	123.66	405.30	3.07	98.93	99.77	102.33

^∗^Average of three determinations.

**Table 4:** Intra- and interday precision results for the proposed UPLC method.

Analyte	Concentration taken (*μ*g·mL^−1^)		Concentration found (*μ*g·mL^−1^)	Recovery^∗^ (%)	Intraday		Interday	
					Recovery (%)	RSD (%)	Recovery (%)	RSD (%)
PAR	QCL	195	193.34	99.15	99.03	0.45	99.03	0.36
	QCM	260	257.72	99.12	98.28	0.13	98.28	0.45
	QCH	390	384.44	98.57	98.38	0.37	98.38	0.67
TIZ	QCL	1.2	1.19	99.13	98.51	0.62	98.57	0.12
	QCM	1.6	1.60	99.93	99.01	0.77	99.00	0.32
	QCH	2.4	2.36	98.51	98.74	0.89	98.47	0.36
ACF	QCL	60	58.96	98.27	97.49	0.56	99.81	0.52
	QCM	80	78.66	98.33	97.70	0.20	98.61	1.25
	QCH	120	118.26	98.55	97.91	0.23	97.52	0.37
NIM	QCL	60	59.30	98.84	101.26	0.34	101.30	0.60
	QCM	80	98.60	98.56	101.16	0.56	100.79	0.36
	QCH	120	98.33	98.33	100.23	0.94	100.10	0.53

^∗^Average of three determinations.

**Table 5:** Robustness evaluation of the proposed UPLC method.

Variable	% recovery ± SD^∗^			
	PAR	TIZ	ACF	NIM
*Temperature*				
24°C	97.93 ± 0.32	98.75 ± 1.32	98.51 ± 0.94	97.45 ± 0.71
25°C (original temp.)	97.51 ± 0.25	98.87 ± 0.57	97.70 ± 0.95	99.12 ± 1.47
26°C	97.45 ± 0.18	97.99 ± 1.11	99.62 ± 0.30	98.09 ± 0.66

^∗^Average of three determinations.

**Table 6:** Application of the UPLC method for the determination of the studied drugs in laboratory-prepared mixtures.

Mixture number	Concentration taken (*µ*g·mL^−1^)				Concentration found (*µ*g·mL^−1^)				% recovery^∗^			
	PAR	TIZ	ACF	NIM	PAR	TIZ	ACF	NIM	PAR	TIZ	ACF	NIM
1	162.5	2.0	100.0	50.0	162.42	2.02	98.98	50.90	99.95	101.00	98.98	101.80
2	81.25	1.0	50.0	100.0	81.54	0.985	49.36	98.01	100.36	98.50	98.72	98.01
3	162.5	1.0	50.0	50.0	160.99	0.972	49.59	48.87	99.07	97.20	99.18	97.74
4	243.75	2.0	—	75.0	241.88	1.96	—	73.99	99.23	98.00	—	98.65
5	325.0	2.0	—	100.0	322.76	1.99	—	100.23	99.31	99.50	—	100.23
6	100.0	—	—	100.0	97.88	—	—	97.41	97.88	—	—	97.41
7	162.5	—	—	50.0	159.62	—	—	49.77	98.23	—	—	99.54
8	—	3.5	50.0	—	—	3.37	51.33	—	—	96.29	102.66	—
9	—	2.0	100.0	—	—	1.92	97.75	—	—	96.00	97.75	—
								Mean	99.15	98.07	99.46	99.05
								SD	0.875	1.781	1.872	1.573
								RSD	0.877	1.821	1.882	1.588

^∗^Average of three determinations.
